# It's all about the children: a participant-driven photo-elicitation study of Mexican-origin mothers' food choices

**DOI:** 10.1186/1472-6874-11-41

**Published:** 2011-09-26

**Authors:** Cassandra M Johnson, Joseph R Sharkey, Wesley R Dean

**Affiliations:** 1 Program for Research in Nutrition and Health Disparities, Social and Behavioral Department, School of Rural Public Health, TAMU 1266, College Station, TX, 77843, USA; 2 Department of Nutrition, UNC Gillings School of Global Public Health, CB# 7461, Chapel Hill, NC, 27599-7461, USA; 3 UNC Center for Health Promotion and Disease Prevention, CB# 7426, Chapel Hill, NC, 27599-7426, USA

## Abstract

**Background:**

There is a desperate need to address diet-related chronic diseases in Mexican-origin women, particularly for those in border region *colonias *(Mexican settlements) and other new destination communities in rural and non-rural areas of the U.S. Understanding the food choices of mothers, who lead food and health activities in their families, provides one way to improve health outcomes in Mexican-origin women and their children. This study used a visual method, participant-driven photo-elicitation, and grounded theory in a contextual study of food choices from the perspectives of Mexican-origin mothers.

**Methods:**

Teams of trained *promotoras *(female community health workers from the area) collected all data in Spanish. Ten Mexican-origin mothers living in *colonias *in Hidalgo County, TX completed a creative photography assignment and an in-depth interview using their photographs as visual prompts and examples. English transcripts were coded inductively by hand, and initial observations emphasized the salience of mothers' food practices in their routine care-giving. This was explored further by coding transcripts in the qualitative data analysis software Atlas.ti.

**Results:**

An inductive conceptual framework was created to provide context for understanding mothers' daily practices and their food practices in particular. Three themes emerged from the data: 1) a mother's primary orientation was toward her children; 2) leveraging resources to provide the best for her children; and 3) a mother's daily food practices kept her children happy, healthy, and well-fed. Results offer insight into the intricate meanings embedded in Mexican-origin mothers' routine food choices.

**Conclusions:**

This paper provides a new perspective for understanding food choice through the eyes of mothers living in the *colonias *of South Texas -- one that emphasizes the importance of children in their routine food practices and the resilience of the mothers themselves. Additional research is needed to better understand mothers' perspectives and food practices with larger samples of women and among other socioeconomic groups.

## Background

Mexican-origin women in South Texas *colonias *(neighborhoods) have some of the highest rates of obesity and diabetes in the U.S. [1], while also maintaining lead roles in their families regarding food and health [2-7]. Existing quantitative studies have provided limited insight into understanding dietary patterns and general food choice for Mexican-origin women/mothers living in a border region like *colonias *[2,8-19]. Previous work for understanding food-related behaviors among Mexican-origin women interpreted behavior based on a prescribed framework and discounted the participants' perspectives [2,6]. Three qualitative studies, which have been published on Mexican-origin women in border areas, have offered context for understanding potential influences on family nutrition and health status [4,7,20], but were not explicitly focused on understanding a mothers' food choices related to food acquisition, preparation, and consumption [3,21,22], activities which are known to influence health outcomes. These observations motivated an examination of mothers' food-related decisions and activities which are critical to improving their own and their children's health. We believe this study is the first of its kind to use a data-driven approach, like grounded theory, combined with participant-driven photo-elicitation (PDPE) to understand how Mexican-origin mothers approach every day food choices in their families.

## Methods

### Approach

Photo-elicitation and photovoice are two methods that rely on a discussion of photographs to learn about the participants' lives from their perspectives, but scholars distinguish photovoice as a group activity (e.g., typically conducted in a series of focus groups) with goals to initiate community action [23,24]. Photo-elicitation is often used in an individual interview [25]. Participant-driven photo-elicitation (PDPE), also described as participatory photo interviewing, benefits from incorporating participatory aspects of photovoice into an individual in-depth interview with participants' photographs [26-30]. Although PDPE overlaps with some photovoice methodology, photovoice is set apart by three goals -- defined by Wang and Burris: 1) to enable people to record and reflect community's strengths and concerns, 2) to promote critical dialogue and knowledge about important issues through group discussion and photographs, and 3) to reach policymakers -- key concepts and methodology [23,31]. The decision to blend PDPE with grounded theory was advantageous in a context-focused study of food choice among Mexican-origin women to involve participants in the research process, share insights and knowledge, capture and understand context [26,28], and potentially to empower participants and facilitate community action [29]. In addition, this approach was well-suited for studying women, families and food [32-34] especially for a study with an immigrant group [35] of Latino or Mexican-origin [33,36,37].

Study protocol and data collection instruments were modified from a study of African American, Hispanic, and non-Hispanic white mothers using PDPE to broadly understand how mothers approached food choice in their families [32,34]. In this study area, most residents are native Spanish speakers [38,39]. Original study materials written in English were adapted for the target population of Mexican-origin mothers living in *colonias *of South Texas. A bilingual, bicultural researcher (native speaker) translated study materials into Spanish, which were reviewed by the *promotoras *(female community health workers from the area) for semantic, conceptual, and normative equivalence and understanding. In addition, a bilingual research scientist native to South Texas examined study materials for acceptability.

### Setting

The *colonias *(neighborhoods) in South Texas are Mexican settlements that form a network of diverse communities along the border [40,41]. *Colonias *are similar to new destination communities of Mexican immigrants which are appearing across the U.S. in interior and rural locations in places previously unsettled by Mexican immigrants [42-45]. These communities are characterized by rapid environmental changes and a dispersed population of mostly immigrants and their families [44,46,47]. Residents experience similar forms of material hardship, including vulnerability to omnipresent environmental exposures and restricted access to infrastructure and basic resources [1,12,20,40,41,48,49] and challenges to health such as limited access to healthy foods, high rates of food insecurity and a disproportionate burden of diet-related chronic diseases [1,13,15,17,48].

**Figure 1:**
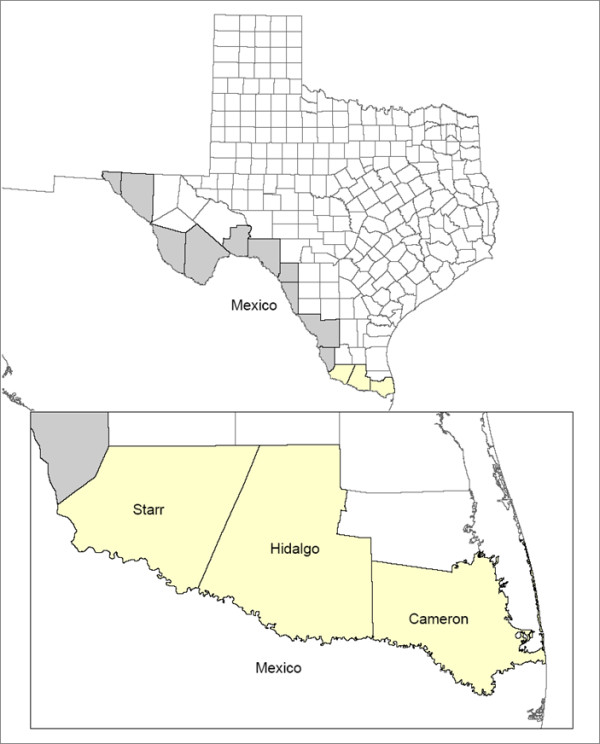
**Map of Hidalgo County, Texas**. This map shows Hidalgo County located along the southern Texas-Mexico border.

Many *colonias *are located in Hidalgo County along the Texas-Mexico border [40], shown in Figure 1. This study was completed in *colonias *near the incorporated towns of San Carlos and Alton, Texas in areas that are considered functionally rural [50]. Census estimates from 2005-2009 indicate that San Carlos and Alton populations were roughly 96- 97% Hispanic/Latino ethnicity, 34-37% foreign-born, with 32-35% families below poverty; the proportion of families in poverty was more than three times the national estimate [38,39].

### Fieldwork Preparation

Research among hard-to-reach residents in *colonias *often relies on *promotoras *who are an integral part of the research team [14]. The four *promotoras *were full-time *promotoras*, native Spanish speakers, and from nearby *colonias *(within a 20 minutes drive by car). These *colonias *were similar in terms of socioeconomic and environmental characteristics, including culture. All women had lived in the area for at least 15 years and were considered part of the community by residents. At the time of the study, the *promotoras' *age ranged from late 30's to 50's, which was similar to the participants' age range (31 years-54 years). The *promotoras *involved with this project served as cultural brokers to ensure the participants' interests were preserved and to represent the participants in conversations with academic researchers. They also were key to recruiting and engaging participants [14,51,52].

For this study, the *promotoras *participated in a half-day training session and pre-testing to prepare for recruitment and data collection. The *promotoras' *training included presenting an overview of the project's goals and activities, discussing the interview guides and potential prompts, conducting an interview, training the participants on how to use a disposable camera, and a question-answer session. There were a few concerns expressed by the *promotoras *including camera use and repetitive story prompts, which resulted in modification of research protocols. In addition to the *promotoras*' training, *promotoras *pre-tested the protocols with two women recruited from the study area, who had similar characteristics as the participants. The women provided informed consent, completed the photography activity, and an audio-recorded in-depth interview with their photographs. They were compensated for their participation in the pre-test activities.

### Recruitment

The *promotoras *recruited a sample of Mexican-origin mothers from the 26 mothers who participated in the 2008 *Colonia *Household Food Inventory (HFI) Project [14]. All HFI participants were native Spanish-speakers. *Promotoras *recruited women who were 1) currently living in *colonias *in the study area, 2) had a working telephone number, and 3) with at least one child under the age of 18 living in the same household. The women were telephoned by *promotoras *and invited to participate in a follow-up study related to mothers and food. The *promotoras *explained the purpose of the project was to understand how mothers approach food choice on a daily basis, including grocery shopping, cooking, and eating. The first 10 participants who were interested and agreed to participate were enrolled into the study. Once mothers agreed to participate, data collection began immediately. During an initial visit, the *promotoras *read the participants the informed consent document and left a hard copy for the participants' records. All participants provided written consent to participate in the study. The Institutional Review Board at Texas A&M University approved this study.

### Data Collection

Two, two-person teams of trained *promotoras *completed an initial visit, where they verbally presented an overview of the study, collected sociodemographic data using a brief one-page survey, and introduced the photography activity, which would generate the participants' photographs. For the photography activity, each participant was provided a 27-exposure disposable camera for taking still photographs and a one-page instruction sheet. The *promotoras *verbally presented the activity, demonstrated how to use the disposable camera, and made a demonstration camera available for the participants to practice taking photographs. Each of the participants was asked to take at least 15 food-related photographs to illustrate their "food experience", defined by day-to-day food choices and activities. Participants were free to photograph anything else with the remaining exposures. The photography assignment was developed to give participants the opportunity to creatively present their perspectives. A few examples were provided (e.g., spending time in your kitchen, anything with food in your family), although the participants were instructed there were no "right" or "wrong" photographs. The *promotoras *explained the photographs should be personally reflective of the mothers and their families. Participants were given approximately one week to complete the photography activity before the *promotoras *collected the cameras for film development. From each camera, two sets of photographs were created. One set was retained for the photo-elicitation interview and the other for the participants to keep for themselves.

After the photographs were developed, *promotoras *scheduled the individual, photo-elicitation interview by telephone. The in-depth interview was conducted in Spanish in the participants' homes. The interview began with *promotoras *asking general questions about the photography activity to obtain participants' reflections about the project. Then, *promotoras *asked the participant to choose six to eight photographs for discussion and encouraged them to select their favorites or interesting photographs. Participants also titled their selected photographs. For each photograph, the *promotoras *used prompts based on the SHOWeD technique. The **SHOW**e**D **acronym represents a set of prompts often used in photovoice and PDPE studies to facilitate critical thinking, reflection, and empowerment; prompts include: ''What do you **S**ee in this picture?'', ''What is **H**appening in this picture?'', ''How does this relate to **O**ur lives?'', ''**W**hy does this problem, concern or strength exist?'' and ''What can we **D**o about it?'' [30,31,33,53]. Interview guide items included two prompts from the SHOWeD technique (e.g., "What do you see in this picture?" and "What is happening in this picture?") and others such as "When was this picture taken?", "What is missing from this picture?", "Why did you select this photograph?", "Is this a typical activity/food for your family?" and "How does this picture make you feel?" Probes such as, "Tell me more about [what's shown in] this picture" and "Explain that further", were used to explore topics in depth. Additional photographs, selected by the *promotoras*, also were discussed to gain understanding about the mother's perspective on food in her family. *Promotoras *selected additional photographs that were not originally selected by the participant, especially those that differed from the majority of her chosen photographs. Each participant was given a $60 honorarium for completing the study.

### Transcription and Translation

Data included mothers' sociodemographic information, participant-generated photographs, and transcripts from participants' photo-elicitation interview. Two bilingual, bicultural researchers used a three-phase transcription and translation process to ensure transcripts' accuracy. Audio files first were transcribed verbatim in Spanish. Then, the Spanish audio files were listened to again to capture any missing words, expressions, emotions, and interview context in Spanish. The verified Spanish text files were translated into English. Finally, the complete English transcripts were reviewed and revised to ensure equivalence. Disagreements regarding translation or meaning were resolved through discussion among the researchers and in conversations with the *promotoras*. This process was used for participants' interviews and a follow-up interview with the lead *promotora *during data analysis. To protect participants' confidentiality, two bilingual, bicultural researchers who assisted in transcription/translation assigned pseudonyms to each participant, based on their audio files and transcripts.

### Grounded Theory

English transcripts from the PDPE interviews were analyzed using a grounded theory approach to understand the lived experience of the participants and to gain contextual insights regarding their food choices [54,55]. Grounded theory does not utilize a preconceived theory to guide the project, but allows insights to emerge from the data using a systematic and creative approach. This approach requires both objectivity, necessary for interpretations to be accurate representations of the data, and sensitivity, necessary to capture nuances in the data [55]. This approach offered a way to amplify the mothers' perspectives regarding feeding their families.

### Data Analysis

This analysis was a textual analysis based on the narratives participants constructed around their photographs, largely omitting discussions focused on the photographs themselves (e.g., how participants decided what to photograph, setting-up the photograph, and personal reflections on the activity) in order to not discount the participants' perspectives regarding the act of photography. Transcripts were read multiple times to capture nuances in the mothers' interviews, and preliminary observations were documented. Summary sheets (memos) were created for each mother that described personal characteristics, key insights, and characteristic quotations that supported emergent findings [56]. Peer debriefing, between coauthors and other researchers involved with the project, was used to discuss initial findings and determine next steps. Research team members brought a range of perspectives and experience; some were native Spanish speakers and of Mexican- or Latino-origin. Transcripts were read again following creation of summary sheets to understand mothers' perspectives and their approach to making food choices. Grounded theory uses a coding process in the following ways: 1) to build theories, 2) as analytical tools for handling large amounts of raw data, 3) to consider alternative explanations, 4) to facilitate a systematic and creative approach simultaneously, and 5) to identify, develop, and relate concepts that are the building blocks of theory [55]. Inductive codes were used to identify most salient portions of text, such as mothers' made-from-scratch cooking, food-related values and creative use of resources, which was initially described as strategies. Initial coding was done manually and interpreted directly by the lead author. Co-authors provided interpretations of these preliminary observations, and observations also were discussed with research team members. At that point, the authors decided the lead *promotora *should be interviewed to provide additional contextual understanding of these observations necessary to continue the analytical process. The lead *promotora *was one of the four interviewers in the project and had several years working with the research team and this community in South Texas. She served as: 1) a cultural broker between participants and academic researchers and 2) a member check to determine if the authors' preliminary observations and interpretations made sense. This interview provided one way to check assumptions and interpretations in order to maintain the objectivity needed to accurately represent the mothers' perspectives [55]. After the lead *promotora*'s interview and several team discussions, transcripts were re-read and inductively coded using Atlas.ti (ATLAS.ti Scientific Software Development GmbH, version 5.7.1, 2011, Berlin, Germany) to better understand the preliminary observations. The transcripts were reviewed to ensure consistency in the application of codes. This additional analysis was used to develop the conceptual framework presented later in results. An iterative process of coding, documentation, and discussion with coauthors and team members was used to identify major themes. Themes then were developed by returning to the data and peer debriefing with coauthors.

## Results

### Participants

Characteristics for the mothers are presented in Table 1. All mothers were born in Mexico and reported annual household incomes less than $10,000. The average age of the participants was 38 years old. None of the mothers were formally employed outside the home, although some mothers described selling food items, such as *tamales *or *dulces *(candy), and half of the mothers' partners were employed. All mothers had at least two children living with them.

**Table 1:** Participants' Characteristics (*n *= 10)

Participant	Age	Education level	Ethnicity	Marital status	Spouse/partner employed	Household composition	Food assistance programs
Carolina	35	9^th ^grade	Mexican/Mexican American	Married	Yes	2 Adults 5 Children	SNAP, WIC, Free/reduced Lunch

Norma	35	12^th ^grade	Mexican/Mexican American	Married	No	2 Adults 3 Children	SNAP, Free/reduced Lunch

Karina	42	9^th ^grade	Mexican/Mexican American	Married	Yes	2 Adults 3 Children	SNAP, Free/reduced Lunch

Berta	37	6^th ^grade	Mexican/Mexican American	Married	No	4 Adults 6 Children	SNAP, WIC, Free/reduced Lunch

Sofia	35	6^th ^grade	Mexican/Mexican American	Married	Yes	2 Adults 5 Children	WIC

Perla	37	9^th ^grade	Hispanic	Not married	No	2 Adults 2 Children	SNAP, WIC, Free Breakfast

Mercedes	31	9^th ^grade	Hispanic	Married	Yes	2 Adults 3 Children	SNAP, WIC, Free Breakfast & Free/reduced Lunch

Anabel	36	9^th ^grade	Hispanic	Living with partner	Yes	2 Adults 4 Children	SNAP, WIC, Free Breakfast & Free/reduced Lunch

Rosalinda	36	6^th ^grade	Hispanic	Married	Yes	2 Adults 3 Children	SNAP, Free Breakfast & Free/reduced Lunch

Alma	54	5^th ^grade	Hispanic	Living with partner	No	2 Adults 2 Children	SNAP, Free Breakfast & Free/reduced Lunch

SNAP = Supplemental Nutrition Assistance Program, WIC = Special Supplemental Nutrition Program for Women, Infants and Children, Free/reduced Lunch = National School Lunch Program, Free Breakfast = School Breakfast Program

Mothers generated more than 200 photographs in the photography activity. On average, mothers took 15 food-related photographs of good quality (e.g., not blurry). Although the photography assignment intended to capture mothers' food experiences, all but two of the mothers took photographs of their children and selected photographs of their children to discuss with the *promotoras*. However, mothers explained their photographs showing food and meals, kitchen appliances, and food activities, such as cooking or washing dishes, were also important to them. The majority of photographs were taken at home. The average interview lasted 73 minutes (730 total minutes for 10 participants; range: 41-117 minutes).

### Themes

With their photographs, mothers provided detailed descriptions of their day-to-day lives and food experiences. Three themes emerged from the data: 1) a mother's primary orientation was toward her children; 2) leveraging resources to provide the best for her children; and 3) a mother's food practices kept her children happy, healthy, and well-fed. These themes characterized the mothers' perspectives on their daily practices and how their perspectives influenced their food practices. The following sections explore each of these themes in more detail.

### Theme 1: A mother's primary orientation was toward her children

From their perspectives, the mothers engaged in endless food-related activities and family care-giving because they were completely dedicated to their children. This primary orientation toward one's children emerged from the transcripts and influenced the mothers' daily practices and routine food decisions and activities. Data supported this observation, with mothers taking many photographs of their children and frequently discussing them in interviews. Their children were of upmost importance to them. In their words, children were everything, a mother's "treasures", and described by one mother as "the engine of my life." Mothers deliberately explained that their day-to-decisions and efforts were motivated by their children. Providing excellent care to their children, including food provisioning, was an essential source of satisfaction and happiness and connected to their identities as mothers. One participant who we named Perla shared: "It fills me up with happiness because...well I love my children a lot and as if [when I am caring] like mother of my children I am doing what it is...to be a mother."

### Theme 2: Leveraging resources to provide the best for her children

**Figure 2:**
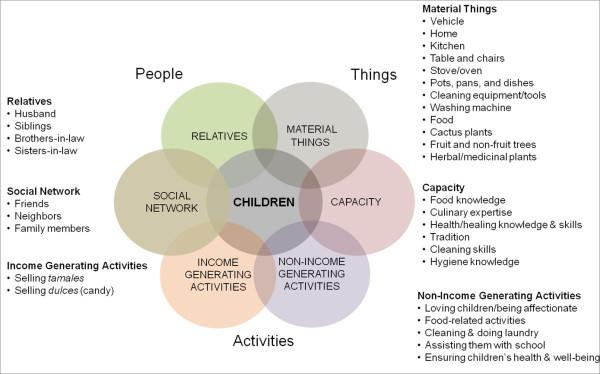
**Conceptual framework**. This figure is the conceptual framework which provides context for understanding a mother's daily food practices.

The second theme described the resources that a mother relied on and integrated to provide the best for the children. This extends the first theme regarding the salience of a mother's primary orientation toward her children. A mother's daily practices were influenced by this primary orientation toward her children and required expert utilization of available resources (e.g., people and things) in creative ways (e.g., activities). Figure 2 presents a conceptual framework based on the data that visually depicts the resources that were critical to understanding a mother's practices. Resources are presented as circular elements. Children are presented in the center to represent a mother's primary orientation toward her children; children were separated from other family members to reflect mothers' perspectives. Six peripheral elements (e.g., material things, capacity, non-income generating activities, income-generating activities, social network and relatives) were categorized broadly into people, things, and activities and arranged around the center circle to show that a mother's primary orientation was toward her children. Placement of peripheral elements around children reflects the participants' perspectives as they discussed leveraging resources in their practices. The peripheral elements are overlapping to acknowledge the complex relationships between a mother's activities and her resources (e.g., people, material things, and capacity), and these elements overlap with the center circle (e.g., children) to illustrate how a mother leveraged resources to provide the best for her children, in spite of a challenging environment.

**Figure 3:**
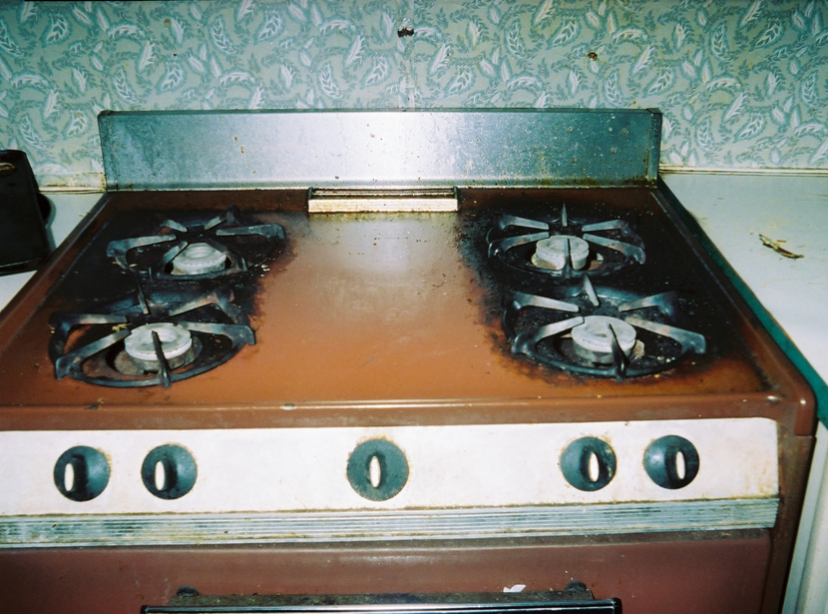
**"My cooking tool or my work tool"**. Carolina took and named this photograph of her stove and commented, "In this one, I see the most important thing. Without her, I do not do anything...Yes, it is one of my most important tools. Without it, even if I wanted to make something for my family, I would not be able to do so if I didn't have it...As I explained to you, without it, I would not be able to make anything for the kids. There are other methods, but they are fast foods that do not have enough vitamins or the nutrition necessary for my family. For example, I would not be able to make a stew...Well, when you guys told me to take pictures of important things for me then I took the picture of the oven because it is important to me and it came to my mind."

*Material things *captured the importance of material possessions that mothers used to provide care to their children and included things inside and outside of their homes. Material things included: a vehicle, the home, a kitchen, stove/oven, pots, pans and dishes, dining table and chairs, outdoor grills, washing machine, other cleaning items (e.g., rags, broom), food, cactus plants, fruit and non-fruit trees, and herbal and medicinal plants. Although money is a material resource, it was not included as mothers did not emphasize it as an important part of providing for their children. Carolina's photograph of her stove is shown in Figure 3. Food items often were described in careful detail to explain the food's meaning and importance to the mother and its role in enabling her to provide for her children. For example, Berta elaborated on a photograph of her pantry and said:

Here, I see my food shelves and pantry at home... This picture is important because it shows the food for the whole family... Well, I took it because... it is my children's food... [I feel] great because I have food to feed them with.

*Capacity *was referred to throughout the transcripts and varied from being implicit to explicit in their discussions. One's ability to manage household responsibilities (e.g., caring for children and household chores) was described implicitly, as was the balancing and timing of food shopping, planning, preparation, and serving associated with eating occasions. One mother indicated that a photograph of her cooking made her feel proud as a mother and signified many things including her culinary expertise -- she shared she had been cooking and making homemade *tortillas *since she was six years old. Mothers did refer to explicit capacity such as food knowledge, culinary expertise, health/healing knowledge and skills, tradition, cleaning skills, and hygiene knowledge. For example, Karina indicated her capacity to bring together different ingredients into a favorite dish (golden brown chicken breasts served with *sopa de arroz *(rice)), which was also fast to prepare and nutritious. She commented, "It is special because it is one of the favorite dishes of the family... It makes me feel happy...That I can prepare it fast and that I know they are going to like it." Mothers demonstrated self-confidence when describing the way such internal resources enabled them to keep their children well-fed, which is discussed later.

*Non-income generating activities *were unpaid activities the mothers engaged in regularly that allowed them to provide excellent care to their children; these included food-related and other activities. The impetus for these activities was their love for their children, and the activities were a source of happiness and satisfaction for the mothers. Common activities included: being affectionate with (or loving) children, bathing them, washing their school uniforms, getting children dressed for school, keeping a clean house, having food ready on clean dishes before and after school, assisting with homework and encouraging children's academic performance. As an example, Berta discussed a photograph of herself preparing eggs, a pot of beans, and heating up made-from-scratch *tortillas *on a *comal *(griddle) and shared, "It makes me feel good...happy...because I do everything for my family." Mothers were able to accomplish these activities by integrating other elements such as capacity and material things with their social network. Activities including protecting and promoting health by integrating their capacity with material things as they treated their children with home remedies made from herbal/medicinal plants and tree fruit and provided children with vitamins from their own fruit trees. Others were oriented around preserving their children's emotional and physical well-being, such as mothers who escorted their children to and from the school bus stop each day to thwart potential kidnapping, offering children a way to refresh themselves from the heat with improvised swimming opportunities and frozen treats, making hand sanitizer available for children, talking to children about the importance of education, and teaching children about relationships and how to love each other. Food-related activities (e.g., meal planning, grocery shopping, cooking, serving, eating, washing dishes, etc.) were motivated by their children as Perla explained here: "I cook because well they are my children. Of course it is my obligation right...Well because they are my children, and I love my family." Data confirmed these activities to be a substantial part of their non-income generating activities. Mothers' food-related activities are discussed later in the third theme.

*Income generating activities *were intense activities that required a mother to integrate all of her tangible and intangible resources to generate income for her family. For example, to make income from selling *tamales*, a mother needed ingredients and equipment; capacity to make *chilé *and meat, wash the leaves, assemble, wrap the *tamales*, market and sell *tamales*; and other individuals (e.g., relatives and social network) to help her sell and buy the *tamales*. Mothers varied in the extent they engaged in such activities. For Anabel, her *tamale *income was the sole source of household income, while Karina discussed selling *tamales *on request, though she made them regularly for her family to consume. It is worth noting that income generating activities imposed additional constraints on a family, such as deciding whether to use income to buy food for the family or for *tamale *ingredients likely to provide income for food and other needs. Another example was Carolina who shared that she sells *dulces *(candy) from her home in what is described as a *tiendita *(little store) and detailed the challenge associated with selling candy while not eating the candy -- She wanted to model healthy habits to her children and likely not diminish her store's profits while maintaining her *tiendita *as a source of income.

*Social network *and *relatives *were related concepts that captured the important individuals who facilitated a mothers' care-giving in both non-income and income generating activities. They are presented separately in the figure to distinguish between family and non-family individuals and to demonstrate the overlap between relatives and one's social network. However, both relatives (e.g., sisters, brothers- and sisters-in-law, and husbands) and other individuals in the social network (e.g., friends and neighbors) provided emotional and physical support that enabled mothers to provide the best care for their children. Mothers relied on other individuals, relatives and non-family members, for many things including transportation to the doctor or to the grocery store, food and health information (used to promote health or to treat children's ailments), an oven to cook their children's meals when their oven was broken, and to watch their children. Relatives and neighbors were often captured in mothers' photographs. To illustrate the relationship between non-income generating activities and the social network, Alma explained how she wakes up at 5:00AM and does not go back to sleep, but attends appointments, cleans her house, and then sometimes goes to clean her sister-in-law's house. This arrangement was better understood knowing that Alma's sister-in-law provides transportation so that Alma can take her son to the doctor; her son has seizures on a monthly basis and requires regular medical care. For many participants (who lived in the area between 4-12 years), they did not have family members other than by marriage in their *colonia*, and relationships with neighbors and friends were critical. Mercedes discussed a photograph where she was making rice for a friend's daughter's *quinciañera *(15^th ^birthday and coming-of-age ceremony for a young woman) and shared that the friends united to make the *quinciañera *possible. She added that, "I have helped many others... It makes me happy because hopefully one day when I need help, they will be there to help me." Although not always the case, two mothers (Karina and Perla) spoke about their husbands who assisted them with the children and in food-related tasks, and Perla described her exceptional husband as a "*Padre Hogareño*" (Home-loving Father). They also discussed the importance of bringing together important individuals with other elements (e.g., material things, capacity, and non-income generating activities) in their income-generating activities, but this relationship was slightly less pronounced. For example, Anabel, who supported her children with *tamales*, relied on her sister to help her sell *tamales *in her sister's *colonia.*

### Theme 3: A mother's food practices keep her children happy, healthy and well-fed

From a mother's perspective, her routine food choices were embedded in her daily practices and influenced by a primary orientation toward her children. Mercedes mentioned the importance of her children always being happy and further explained her priorities in food choices:

For me I am always glad that my kids tell me they are hungry because I want to make sure they eat...because my youngest daughter...will tell me, 'I don't like that' or 'it was nasty' and she didn't eat and will ask me for food. She will tell me she didn't eat anything at school the whole day, and so that is why she gets home and eats... I took this picture so they can see that she is eating...Well I cook deliciously and what they like...Yes, for them to eat well so they don't complain that they are hungry shortly after eating. I want them to eat well.

Their food practices supported mothers' priorities to have happy, healthy, and well-fed children. The term "practices" is used here in place of "strategies" to distinguish between discrete activities used to mitigate acute food-related hardship and routine activities that were embedded in their daily lives. These practices involved a mother bringing together many resources (e.g., foods particularly meat proteins, kitchen appliances and tools, culinary expertise, and important individuals) in creative ways to overcome challenges associated with not having large amounts or varieties of food. A quotation from Carolina captures a mother's perspective on important elements in her food practices and her priorities:

Just as the family and the stove, everything is important to me...All of them. There is not one [that] is specific. I could tell you that it is my family but the stove is also, because without this tool, without the dishes, my kids would not be happy and I would not be able to feed them what they need.

Referring to the conceptual framework, food practices bring together elements on the right-hand side: material things, capacity and non-income generating activities. A cultural belief guided their practices, as one mother explained: "*Lo que come uno comen los demas. Comemos todos*" or "What one eats everyone else eats. We all eat." Her quotation was a derivative of an expression in Spanish "*donde come uno comen dos*," or "where one eats two eat" representing a cultural belief that even in times of food-related hardship there is always something to make to eat [57]. However, mothers described how their efforts went beyond providing enough food for everyone to be satisfied, including the mother.

**Figure 4:**
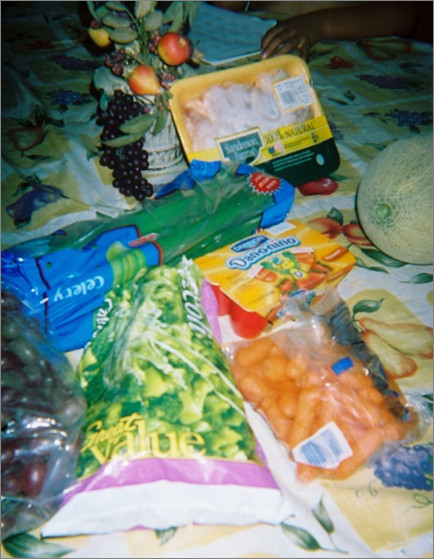
**"Their favorite soup"**. Sofia took and named this photograph of foods purchased at the store-frozen broccoli, fresh baby carrots, celery, cantaloupe, individual yogurts and fresh chicken. She commented: "Well this is what I am going to prepare for them during the day...it is a dish they asked me for, primarily, that they wanted to eat, so I made it. [It's for] a soup, but I didn't put much chicken in it. Because they don't like chicken that much. They like vegetables more than anything like carrots, celery, broccoli...They tell me, 'Thank you, Mommy for making us this to eat'...Every time I make something for them to eat, they thank me."

Mothers detailed the following reasons why they engaged in an endless cycle of cooking, feeding, and cleaning: 1) to make their children happy by continually providing foods their children liked to eat and accommodating children's special requests, 2) to offer their children the best foods which were nutritious, tasty, and better quality than food prepared by other individuals or purchased elsewhere (e.g., "in the streets" (mobile food vendors) and in *pulgas *(flea markets) [48,58], and 3) to ensure their children were healthy and nourished (e.g., not hungry) so they could do well in school and have a better future. All three of these motivations were connected to a mother's love for her children and her satisfaction in providing the best care and foods to her children. It is important to note that though participants described the importance of their children eating to stay nourished, they strongly emphasized the importance of their children's happiness and satisfaction. In other words, mothers wanted their children to enjoy the foods they prepared for their children. Figure 4 shows Sofia's photograph of the ingredients in her children's favorite soup and emphasized various values, including her own satisfaction. Mothers' most salient values were bidirectional satisfaction (outcome when children liked the foods their mother prepared and a mother's happiness in providing such foods to her children), children's nourishment (providing foods to meet basic need for food or limit hunger), and nutrition (specific health benefits associated with food including "good", low-fat foods). Other values including taste (the flavors of the food), quality (the superior nature of a mother's homemade food compared to foods made by others), health (the physiological benefits associated with eating certain foods), variety (of dishes and accompaniments offered at eating occasions and variety of meals) and tradition (cultural and family practices that were incorporated and sustained through mother's food activities) were less emphasized among all mothers, but nonetheless significant to individual mothers. These values were discussed in context of a mother's exceptional care-giving to her family.

This sample of mothers provided many examples of their extensive made-from-scratch cooking, which often started early in the morning (e.g., 5 or 6AM) and continued through a late evening meal or snack. These activities were scheduled around children's school bus pick-up times in the morning and drop-off times in the afternoon, as mothers desired to provide each child with nourishing breakfasts in the morning and to promptly address each child's hunger after school. In addition, conversations contained detailed descriptions of food practices including recipes, where the mothers shared their expertise and experiences with the *promotoras*. While elaborating on foods shown in their photographs, a mother's abilities, especially her food knowledge and culinary expertise, were quite apparent. Many conversations focused on how they created delicious, hand-crafted *tortillas*, *gorditas*, and *tamales *for their families. Although some of the mothers utilized just-add-water *tortilla *and *masa *mixes and one mother used a *gordita *press, foods typically were made-from-scratch with very little prepared foods. Most meals included several made-from-scratch components including beans, *sopas *(soups) or *caldos *(broths), and *salsas*. One mother Karina proudly shared a photograph where she made all of her children's favorite dishes during the same occasion because she wanted to make all of her children happy, which also made her happy as a mother.

**Figure 5:**
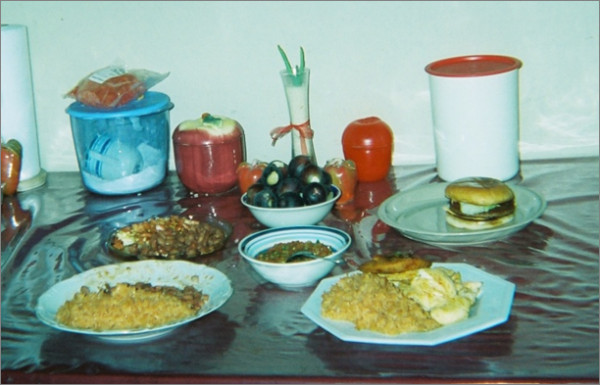
**"My kids' favorite foods"**. This photograph was taken and named by Karina, who prepared each of her children's "favorite" dishes. She said: "I had to do something different and then the hamburger...[the] beans...I took that picture that day because I asked myself what I was going to make for them and then I thought I could make them all each of their favorite dishes, what each of them likes, and I took the picture and that was it."

Specific and salient food practices included rotating meals, refashioning leftovers, preparing coupled dishes, and creating a meal from several, smaller dishes, which helped ensure that multiple children would be nourished and satisfied simultaneously and also allowed mothers to be satisfied in their efforts to provide the best foods for their children. Several mothers shared how they either rotated meals on a regular basis or maintained a schedule of meals to maximize variety and keep their children well-fed. Another reason for rotating meals was to balance more traditional, less healthy meals such as *enchiladas *with healthier meals such as baked chicken that were prepared more frequently. A few participants explained how they refashioned a leftover food item to stretch their food budget and provide children with foods they liked to eat. One participant said, "...You can try, so they can continue eating...[For example] the beans-they hardly ate it, but I combined it with *sopa de arroz *(rice), and they ate it that way." Another example was using a slow-cooked, seasoned meat originally made for *tamales *for making a new dish, such as meat-filled *gorditas*. In most descriptions of mothers' cooking, foods were rarely made in isolation. Figure 5 shows Karina's photograph of all her children's favorite foods. The following quotation demonstrates her simultaneous approach to preparing several dishes. She said "I was finishing up one of the dishes, and I was thinking on what else I could make, from the ones that I had made in that bit of time..." Mothers described preparing coupled dishes to maintain efficiency in food preparation and preserve valuable sources of meat protein; coupled dishes were those that shared a meat protein or another base ingredient and were prepared together. For example, one mother outlined how she: 1) boiled bone-in chicken to use some of the meat in a dish called *molé *(chicken served with homemade *molé *sauce); 2) used the protein-rich broth (from the chicken) to make *sopa de arroz *(rice); and 3) planned to use the balance of leftover chicken for another dish such as *flautas*. In addition, mothers spoke about combining several smaller dishes into one meal, which was perfectly captured in Norma's quotation:

And each dish should not be in large quantities, no. If you make a little and a little of each, or rather everything, you make it [happen] and many people will be happy...And with [a few] small portions, but from one, you can get several [portions]...and everyone eats...There is always something to make...Because let's say that you have, potatoes, eggs and beans, from there you make a dish...

The reasoning for combining several small dishes together into one meal was the same as for the other practices -- mothers could always make something for all of their children to eat and enjoy. This was supported by another mother who said "this is not a lot" to explain how her dishes were not large (amounts of food) on their own, but in combination with others, she was able to provide enough food to feed her family.

In addition, mothers were confident in their daily food practices and ability to keep their children well-fed, even in an environment with persistent child-level food insecurity [48]. Several participants indicated they took photographs because they were pleased with the foods they made and their photographs (e.g., "Well, this picture, I liked the way it came out!") and wanted to share their photographs with the *promotoras *(e.g., "I like it, and I wanted to show them to you for being part of this program to show what I do"). Mothers used photographs to demonstrate self-confidence in two ways: 1) providing their children with enough foods, nutritious foods, and most importantly, foods their children liked to eat, and 2) making more challenging, traditional Mexican dishes, such as *gorditas *and *tamales*. Mothers demonstrated self-confidence with statements such as this one from Berta: "...Because I am the one that makes it...because I am the [one] in charge in the kitchen...[I'm] very happy...because I make the foods and I can feed my family." They were pleased with their cooking abilities and proud because they knew their children preferred their foods and would "devour it" or eagerly consume the food. A few mothers also expressed pride in making certain challenging, traditional dishes such as *gorditas *and *tamales*. Their self-confidence bolstered the capacity present in their daily food practices. Norma explained her photograph of *tamales *made her: "Very happy...There are a lot of people that want to make them, but... Sometimes, I want to make something new, but it doesn't come out. But, I know that I do know how to make *tamales...*" Lastly, a few mothers also used their food practices to creatively express themselves (e.g., "playing" with different vegetables and mixing sweet and salty tastes).

## Discussion

This PDPE study makes novel contributions to the literature by describing Mexican-origin mothers' perspectives on their daily practices, priorities, and food choices in an integrated manner, which has been missing from nutrition and health literature [2,4-7,59]. In doing so, these findings provide valuable context and a conceptual framework for better understanding dietary patterns and food practices of low-income Mexican-origin mothers. For example, this analysis revealed a common belief in Latino cultures "*donde come uno comen dos*" or ("where one eats two eat") [57] that has been overlooked in the literature regarding food choice coping strategies among Mexican-origin families [5], but has implications for food security and nutrition research.

Our general finding that mothers' food practices in particular were influenced by their roles as mothers is supported by related literature about women of Mexican-origin [2-4,60,61] and of other ethnocultural backgrounds [21,62,63]. Prior work has reported that Mexican-origin and Latina women consider their food-related roles to be an integral part of their identities [2-5], which these data confirmed. The observation that mothers were primarily responsible for ensuring their families' physical, emotional, and spiritual health [3,6,64] and "the one in charge" of family food choices [3,60,61] has been well-documented by others. Pinto and Coltrane suggested that Mexican immigrant mothers may have more responsibility for domestic activities because they had lower incomes and less education, worked fewer hours, and had more family members at home, when compared to women who had higher incomes [61]. Our finding for mothers living in *colonias *may not be the case for middle- and upper-class Mexican-origin and Latina women. In any case, mothers' routine activities related to food and health are critical given the prevalence of chronic disease observed in Mexican-origin women [1,20] and children along the U.S.-Mexican border and nationwide [65-67].

Mothers' food practices highlighted salient values, which were influenced by a primary orientation toward their children and already have been identified as important values in mothers' food choices [5,68]. However, these mothers went beyond basic nourishment and stressed the importance of preparing homemade foods that would keep their children happy, healthy, and eating well. This particular combination of values may be most noticeable because mothers considered this to be part of their identities as mothers [5]. Specifically, research suggests that providing satisfying food and treating children with made-from-scratch foods may be part of a parental identity among poor Latino families [5]. Also, mothers may have treated their children with made-from-scratch foods as a way to alleviate hardships associated with poverty [20]. A study of Latino families suggested that parents dealing with unrelenting poverty and food insecurity wanted to satisfy their children because it is "an achievable source of gratification," however they mentioned that satisfying children with food can result in parents providing children with "unhealthy foods" [5].

Results from the current study should be considered in the context of material hardship, including the lack of basic housing, transportation, food insufficiency and food insecurity, and access to health/medical care that is prevalent in many *colonias *[49]. Specifically, the literature supports our observations of various and persistent stressors (e.g., sociocultural, financial, and environmental) [20], which mothers attempted to mitigate as they preserve their children's physical and emotional health and well-being. Their family care-giving activities, including food-related, may have been a way to cope with daily stressors attributed to a challenging environment [20]. Unfortunately, there is very little literature that discusses the capacity of low-income Mexican-origin women living in border or other underserved areas, and practices employed in care-giving or food provisioning [59]. Related literature does suggest that low-income women and mothers utilize a range of food choice strategies, or every day practices as observed here, to reduce hardships associated with food insecurity [5,69,70]. In a study of diverse Latino families in a relatively small metropolitan area, a Mexican mother explained how she dealt with inadequate benefits with, "where three eat, the fourth can, too" [5], which was similar to these mothers.

Lastly, this analysis highlighted that the participant-mothers were "*reinas de la cocina*" (queens of the kitchen), which supports Abarca's description of Mexican-origin women and mothers being "cooks-as-artists" whose efforts kept their children well-fed and eating *con gusto *(with much enjoyment) [4]. Abarca used the term "cooks-as-artists" to demonstrate how the women/mothers viewed the kitchen as their *space *(original emphasis retained) to express themselves, demonstrate agency or affirm themselves as mothers and "cooks-as-artists", and to take care of their families [4]. This was supported by González and others who describe a salient characteristic of *la mujer Mexicana *(the Mexican woman) as one who embodies "the right to claim a space for creating agency and cultural integrity" [71-74]. Related observations have been made by Dean and colleagues who noted that female Hispanic participants did not describe managing food-related responsibilities and constraints as "drudgery", as suggested by other writings on the gendered nature of food provisioning, but as "creative work" [3].

### Implications

Given that the participant-mothers lived in communities which are similar to new destination communities, this work provides insights relevant to researchers, practitioners, and policymakers at regional and national levels. This analysis found that Mexican-origin mothers articulated several distinct priorities for their children, which required them to protect their children against continual threats to their physical and emotional health and well-being. There are several implications associated with these findings. First, nutrition *per se *might not be a critical priority for mothers, but mothers emphasized priorities that were well-suited for encouraging improved eating behaviors. Second, these mothers were primarily responsible for their family's needs for food, medical care, and transportation with limited assistance from spouses. These additional constraints may be detrimental to a mother's health. Although not the focus here, mothers communicated anxiety about "struggling" with material hardship and reconciling what researchers describe as "competing demands" [20,64]. For example, Anabel mentioned that although her *tamale *income provided for her children, sometimes she did not have money to buy *tortillas *for her family. This finding regarding the influence of stressors on the mothers' health is particularly important given that many Mexican-origin women have poor physical and mental health, especially true for many women in border *colonias *[1,20,59]. In addition, mothers may not place a priority on their own health. As Carolina explained, her children were "the most important thing for me. If they are well, I am well." It is possible that mothers do not consider their own health as essential for ensuring their children's health. Third, mothers emphasized the importance of providing foods that their children liked to eat, which posed additional challenges for those working to improve diet and diet-related health outcomes in this population. Fourth, mothers' food practices were guided by the belief that "there is always something to eat." This belief has important implications for those who measure food security and suggests the prevalence of food security in areas like *colonias *is underestimated [48]. Lastly, mothers demonstrated agency and creativity through their food practices in their pursuit of happy, healthy children in spite of adversity. The resilience observed by this sample of Mexican-origin mothers and documented by others may help better address health disparities in Hispanic subgroups [20,47,75-77].

### Strengths

Findings from this project: 1) address methodological limitations in previous work for understanding food-related behaviors among Mexican-origin women, such as interpreting behavior based on a prescribed framework and discounting the participants' perspectives [2,6] and 2) advance the field by using PDPE to understand food and health practices relevant for mothers' and children's nutrition and overall health [32-34]. Strengths primarily derive from advantages associated with combining participant-driven photo-elicitation (PDPE) with grounded theory, which allowed us to: 1) overcome limitations associated with literacy, cultural barriers, and traditional interviews, 2) produce different type of data, and 3) capture and preserve the context surrounding routine food choices [26,28,29,54,55]. However, it is worth noting this project was guided by cultural humility, or a respectful and honoring attitude [78] that seeks to understand "cultural issues intermixed with health issues," and by developing relationships with community members, participants, and *promotoras *over time [79]. This project benefited tremendously from the *promotoras*, who were trusted by the participants and valued members of the research team [14]. The quality of data produced in the study spoke to the relationship between the *promotoras*, the community, and this group of mothers. With this participatory and innovative approach, participant-mothers reflected on their photographs, shared their daily food experiences and affirmed their abilities to sustain and nurture their families through detailed descriptions of daily food practices.

### Limitations

Although this work has many strengths, this study was not without limitations. First, analysis relied on English transcripts only, which is a methodological weakness. Original data were in Spanish, and despite rigorous efforts to preserve equivalence in the English translations, some meaning may have been lost. Future work would benefit from parallel analyses in English and Spanish and discussion of data interpretation amongst researchers. Second, the sample was a relatively small group of mothers living in two disparate areas of Hidalgo County and does not necessarily represent Mexican-origin mothers living in other areas. These mothers may have been "exceptional" mothers who engaged in non-typical food behaviors, such as preparing made-from-scratch meals. Although the p*romotoras *did not advertise the study as being focused on mothering, nutrition, or health, more devoted or health-conscious mothers could have self-selected to participate in the study. Lastly, social desirability may have influenced the participants to want to participant and please the *promotoras *in their responses; however, having an established relationship with the participants also allowed the *promotoras *to probe further in the interviews and obtain richer data.

## Conclusions

Findings from this paper establish that this sample of Mexican-origin mothers demonstrated a primary orientation toward their children that influenced their daily food practices. Mothers leveraged their resources and employed specific food practices to ensure their children were happy, healthy, and well-fed every day. Considering the challenges mothers described, they demonstrated extraordinary agency and creativity in their practices. Findings suggest that mothers' practices incorporated some healthy behaviors that may reduce overweight and diabetes in their family. Future projects may benefit from developing programs that incorporate a mother's priorities to have happy, healthy, and well-fed children and encourage her to continue healthy practices. In addition, public health efforts may be able to improve mothers' health by encouraging them to focus on their own health so they can continue to provide the best for their children. Additional research is needed to better understand mothers' perspectives and practices with larger samples of mothers and among other socioeconomic groups.

## Competing interests

The authors declare that they have no competing interests.

## Authors' contributions

CMJ and JRS conceived and designed study. CMJ analyzed data. CMJ, JRS, and WRD contributed to data interpretation. CMJ prepared first draft. CMJ, JRS, and WRD made revisions, and all authors read and approved final manuscript.

## Pre-publication history

The pre-publication history for this paper can be accessed here:

http://www.biomedcentral.com/1472-6874/11/41/prepub
